# Supplementary material for: A covalent PIN1 inhibitor selectively targets cancer cells by a dual mechanism of action
Source: Nat Commun. 2017 Jun 9;8:15772. doi: 10.1038/ncomms15772 (PMC5472749; doi:10.1038/ncomms15772)
Supplement: Supplementary Information — Supplementary Figures, Supplementary Tables, Supplementary Methods and Supplementary References [file ncomms15772-s1.pdf]

Supplementary Figures

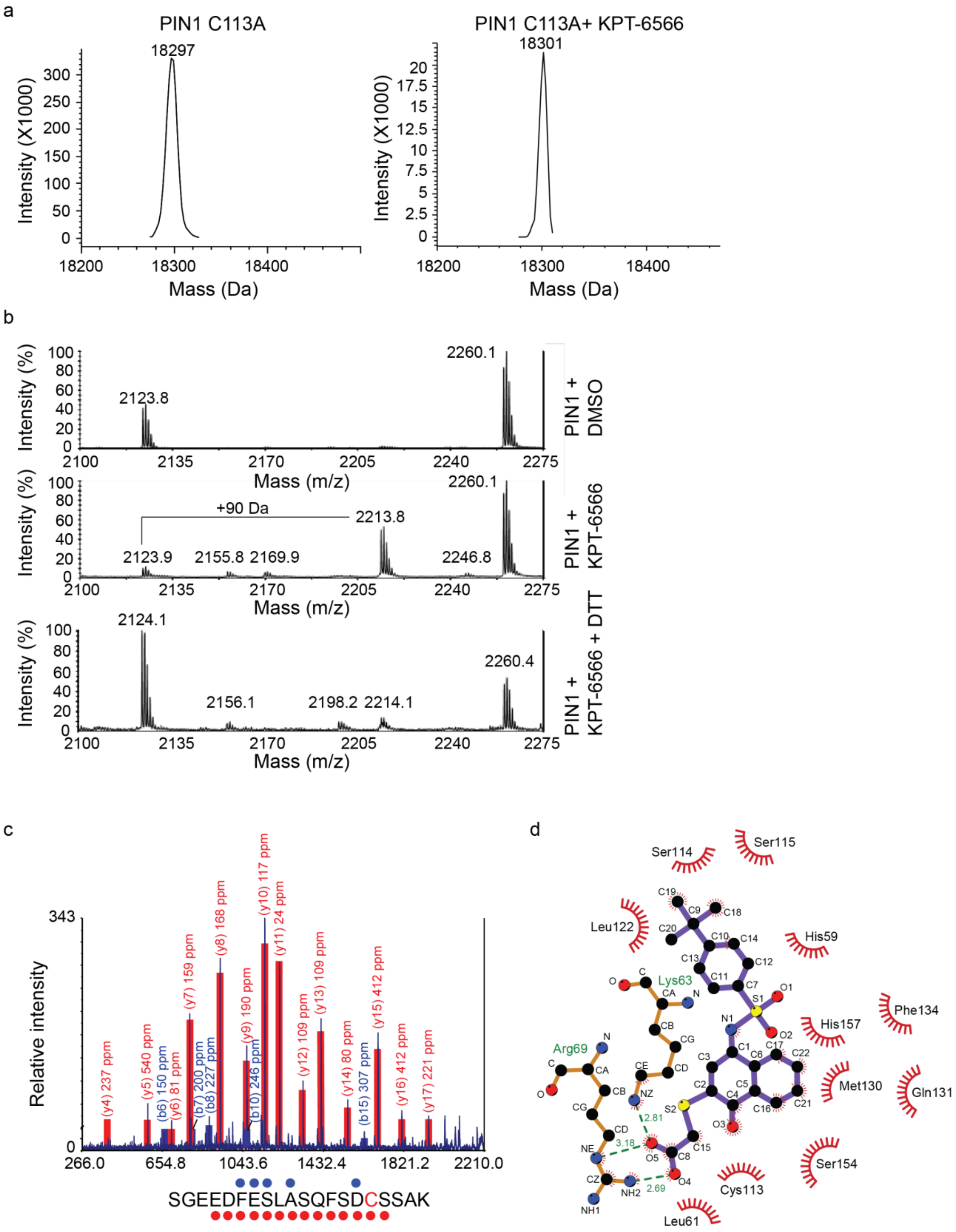

e

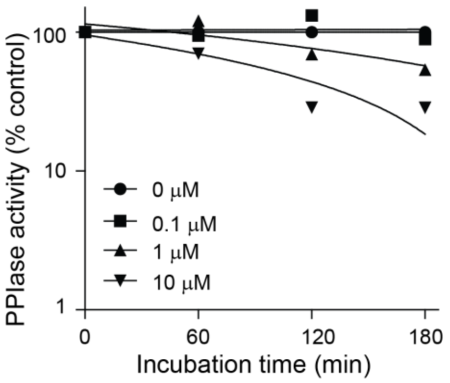

f

```
CLUSTAL O(1.2.1) multiple sequence alignment


sp|P62937|PPIA_HUMAN      -----------------------------------------------MVNPTVF------ 7
sp|Q13526|PIN1_HUMAN      ------------------------------------------------------------ 0
sp|Q02790|FKBP4_HUMAN     MTAEEMKATESGAQSAPLPMEGVDISPKQDEGVLKVIKREGTGTEMPMIGDRVFVHYTGW 60

sp|P62937|PPIA_HUMAN      -----------------------------FDIAVDGEPLGRVSFELFA--------DKVP 30
sp|Q13526|PIN1_HUMAN      ------------------------------------------------------------ 0
sp|Q02790|FKBP4_HUMAN     LLDGTKFDSSLDRKDKFSFDLGKGEVIKAWDIAIATMKVGEVCHITCKPEYAYGSAGSPP 120

sp|P62937|PPIA_HUMAN      KTAENFRALSTGEKGFGYKGSCFHR-----IIPGFMCQGGDFTRHNGTG-----GKSIYG 80
sp|Q13526|PIN1_HUMAN      ------------------------------------------------------------ 0
sp|Q02790|FKBP4_HUMAN     KIPPN-ATLVFEVELFEFKGEDLTEEEDGGIIRRIQTRGEGYAKPNEGAIVEVALEGYYK 179

sp|P62937|PPIA_HUMAN      EKFEDENFILKHTG--------PGILSMANAGPNTNGSQFFI--CTAKTEWLDGKHVVFG 130
sp|Q13526|PIN1_HUMAN      --------------MADEEKLPPGWEKRMSRS---SGRVYYFNHITNASQWERPSG-NSS 42
sp|Q02790|FKBP4_HUMAN     DKLFDQRELRFEIGEGENLDLPYGLERAIQRMEKGEHSIVYL----------KPSY-AFG 228
                                                 *     .     .    ::            .    .

sp|P62937|PPIA_HUMAN      KV-KEGMNI--------------------------VEAME-------------------- 143
sp|Q13526|PIN1_HUMAN      SGGKNGQGEPARVRCSHLLVKHSQSRRPSSWRQEKI------------------------ 78
sp|Q02790|FKBP4_HUMAN     SVGKEKFQIPPNAELKYELHLKSFEKAKESWEMNSEEKLEQSTIVKERGTVYFKEGKYKQ 288
                          .  *:

sp|P62937|PPIA_HUMAN      ----------------RFGSRNGKT----------------------SKKITIADCGQLE 165
sp|Q13526|PIN1_HUMAN      ----------------TRTKEE-----ALELINGYIQKIKSGEEDFESLASQFSDCSSA- 116
sp|Q02790|FKBP4_HUMAN     ALLQYKKIVSWLEYESSFSNEEAQKAQALRLASHL--NLAMCHLKLQAFSAAIESCNKAL 346
                                             ..:                         :    : .* .

sp|P62937|PPIA_HUMAN      ------------------------------------------------------------ 165
sp|Q13526|PIN1_HUMAN      --KARGDLGAFSRGQMQKPFEDASFALRTGEMSG--PVFTD------------SGIHIIL 160
sp|Q02790|FKBP4_HUMAN     ELDSNNEKGLFRRGEAHLAVNDFELA--RADFQKVLQLYPNNKAAKTQLAVCQQRIRRQL 404

sp|P62937|PPIA_HUMAN      -------------------------------------------------------      165
sp|Q13526|PIN1_HUMAN      RTE----------------------------------------------------      163
sp|Q02790|FKBP4_HUMAN     AREKKLYANMFERLAEEENKAKAEASSGDHPTDTEMKEEQKSNTAGSQSQVETEA      459
```

Supplementary Figure 1. (**a**) Mass spectrum deconvolution of DMSO treated (left) and KPT-6566 treated (right) PIN1 mutant C113A. (**b**) Mass spectra of trypsin digested DMSO (top), KPT-6566 (middle), and KPT-6566 + DTT treated PIN1 (bottom) in the region 2100-2276 m/z. A clear shift of 90 Da was observed in the peptide 2123.9 Da corresponding to SGEEDFESLASQFSDCSSAK peptide upon KPT-6566 treatment and was absent in DTT co-treated sample. (**c**) MS/MS spectrum of the 2213 m/z peak. Sequence is correctly assigned to the peptide SGEEDFESLASQFSDCSSAK bearing modified C113 (highlighted in red). Fragment ions containing the peptide C-terminus (y-type) or N-terminus (b-type), along with the associated mass errors are shown in red and blue, respectively. (**d**) 2D image showing hydrophobic contacts between KPT-6566 and PIN1. (**e**) Semi-logarithmic plot of PIN1 PPIase activity in presence of increasing concentrations of KPT-6566 at different incubation times, obtained in PPIase assays. Results are indicated as means of three independent experiments. (**f**) Protein sequence alignment obtained with CLUSTALW. PPIase domains are highlighted in grey. Cysteine residues (red) are highlighted in yellow.

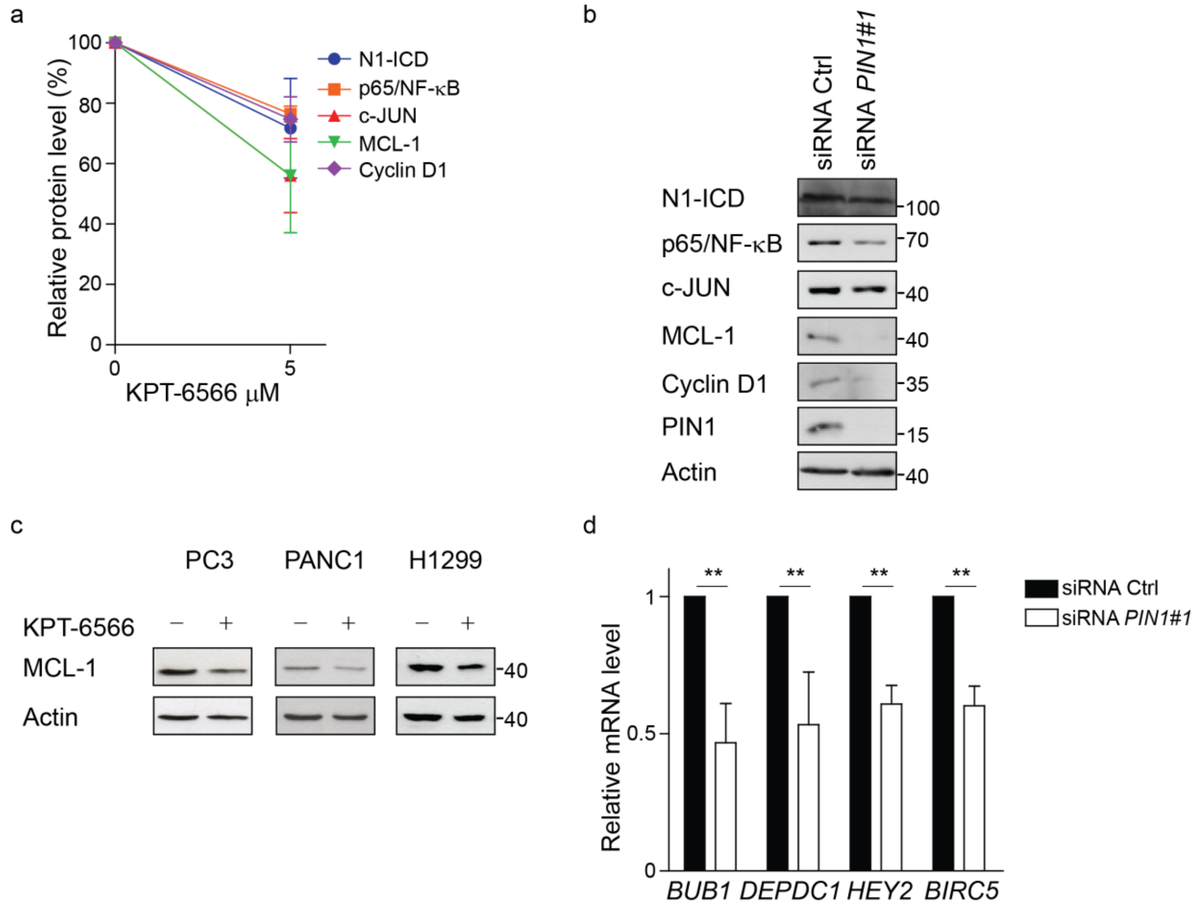

Supplementary Figure 2. (**a**) Graph showing protein quantification of immunoblotting of different PIN1 client proteins in MDA-MB-231 cells treated with KPT-6566 or DMSO for 48hrs. Quantification was obtained with ImageJ software. Protein levels were normalized to actin. (**b**) Immunoblotting of the indicated proteins from MDA-MB-231 cells transfected with control siRNA (siRNA Ctrl) o with *PIN1* siRNA (siRNA *PIN1#1*). (**c**) Immunoblotting of MCL-1 from PC3, PANC1, H1299 cells treated with 5 μM KPT-6566 (+) or DMSO (-) for 48hrs. (**d**) Quantitative RT-PCR analysis in control (siRNA Ctrl) or *PIN1* silenced (siRNA *PIN1#1*) MDA-MB-231 cells. **b**, **c** actin levels are reported as loading control; size markers are indicated. Data shown in **a** and **d** are the means ± s.d. of n=3 independent experiments, $**P<0.01$; two-tailed Student's *t*-test.

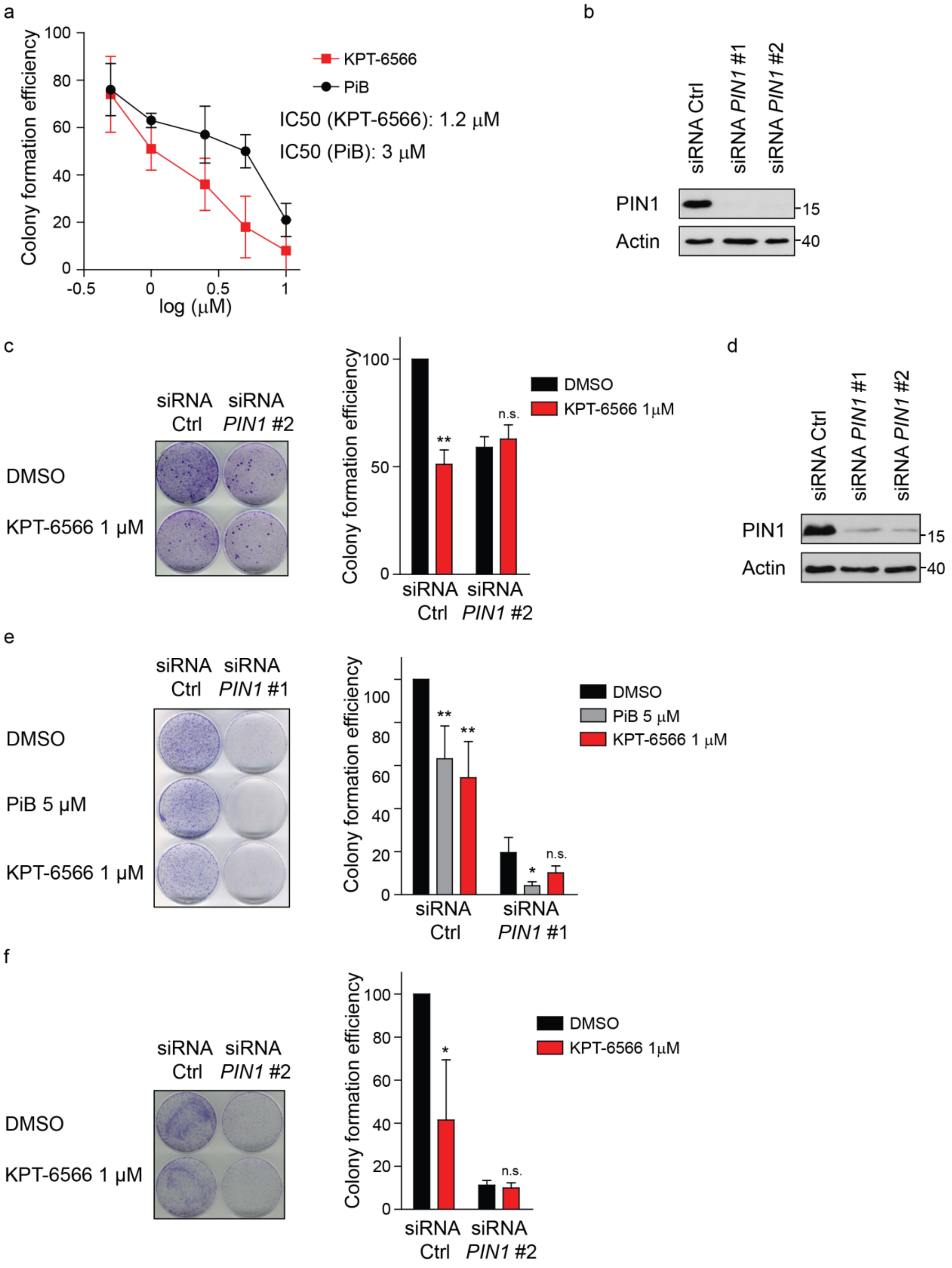

a
Colony formation efficiency
log (μM)
KPT-6566
PiB
IC50 (KPT-6566): 1.2 μM
IC50 (PiB): 3 μM
b
siRNA Ctrl
siRNA PIN1 #1
siRNA PIN1 #2
PIN1
Actin
15
40
c
siRNA Ctrl
siRNA PIN1 #2
DMSO
KPT-6566 1 μM
Colony formation efficiency
DMSO
KPT-6566 1μM
n.s.
d
siRNA Ctrl
siRNA PIN1 #1
siRNA PIN1 #2
PIN1
Actin
15
40
e
siRNA Ctrl
siRNA PIN1 #1
DMSO
PiB 5 μM
KPT-6566 1 μM
Colony formation efficiency
n.s.
f
siRNA Ctrl
siRNA PIN1 #2
DMSO
KPT-6566 1 μM
Colony formation efficiency
DMSO
KPT-6566 1μM
n.s.

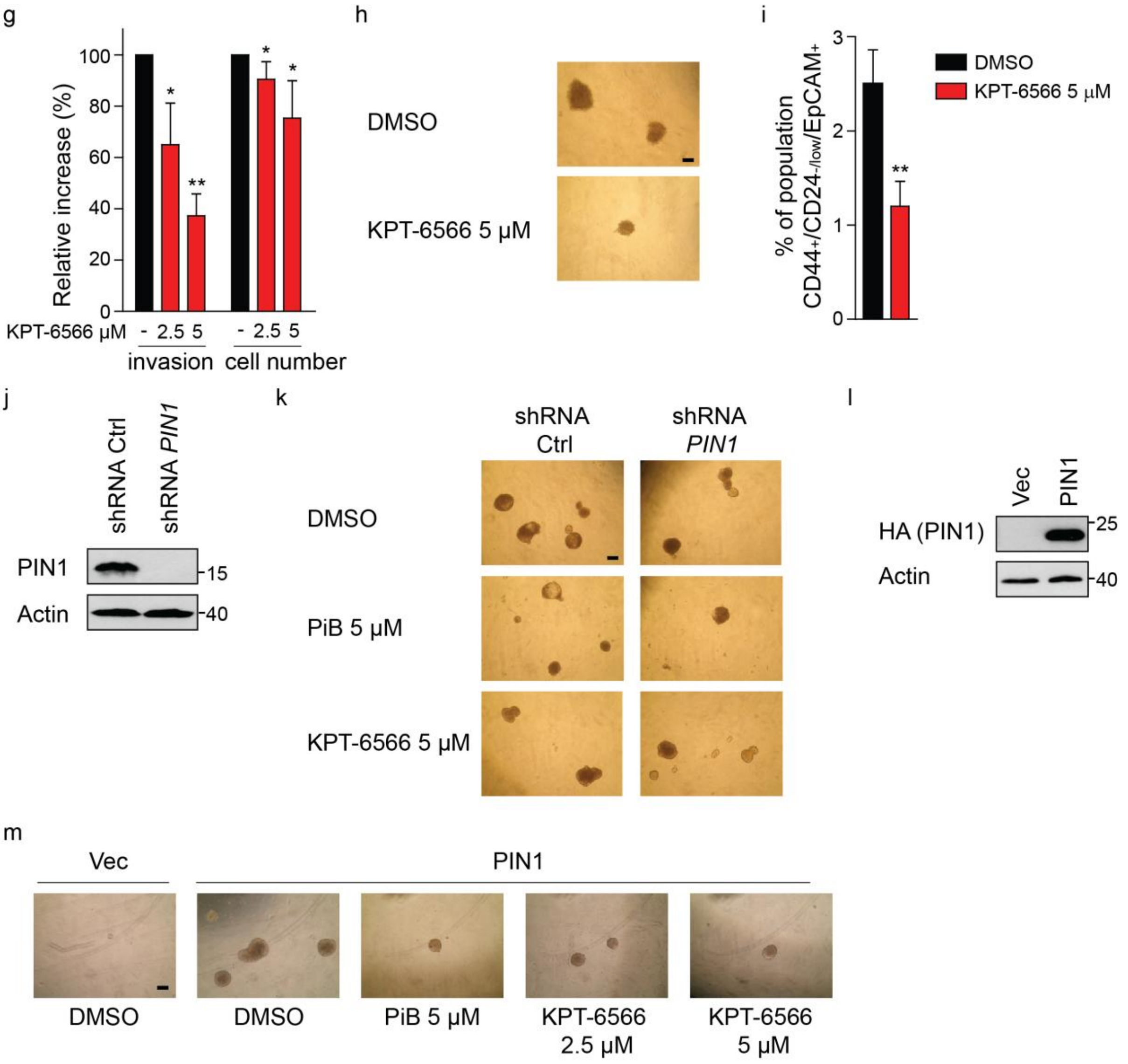

Supplementary Figure 3. (**a**) Graph showing colony formation efficiency of MDA-MB-231 cells treated with DMSO or increasing concentrations of PiB, KPT-6566. IC50 of both drugs are indicated. (**b**) Immunoblotting of the indicated proteins in cell lysates from MDA-MB-231 cells in the indicated experimental conditions. (**c**) Left, representative pictures of MDA-MB-231 colonies in the indicated experimental conditions. Right, histogram showing colony formation efficiency of MDA-MB-231 cells in the indicated experimental conditions. (**d**) Immunoblotting of the indicated proteins in cell lysates from PC3 in the indicated experimental conditions. (**e**), (**f**) as in **c** for PC3 cells. (**g**) Histogram showing invasive ability and proliferation (cell number) of PC3 cells plated in Matrigel-coated Boyden chambers in the indicated experimental conditions for 20hrs. (**h**) Representative images of secondary mammospheres obtained with MDA-MB-231 cells in the indicated experimental conditions. Scale bar (200 µm) is indicated. (**i**) Histogram showing percentage of $CD44^{+}/CD24^{-/low}/EpCAM^{+}$ MDA-MB-231 cells in the indicated experimental conditions. (**j**) Immunoblotting of the indicated proteins from MCF10AT1 cells transduced with

control- (shRNA Ctrl) or *PIN1* shRNA (shRNA *PIN1*) expressing vectors. (**k**) as in (**h**) for MCF10AT1 cells. (**l**) Immunoblotting of the indicated proteins from MCF10A cells transduced with empty- (Vec) or HA-PIN1 over-expressing vectors. (**m**) as in (**h**) for MCF10A cells. **b**, **d**, **j**, **l**, actin levels are reported as loading control, size markers are indicated. Data shown in **a**, **c**, **e**, **f**, **g**, **i**, are the means ± s.d. of n=3 independent experiments, **P*<0.05, ***P*<0.01, n.s. not significant; two-tailed Student's *t*-test.

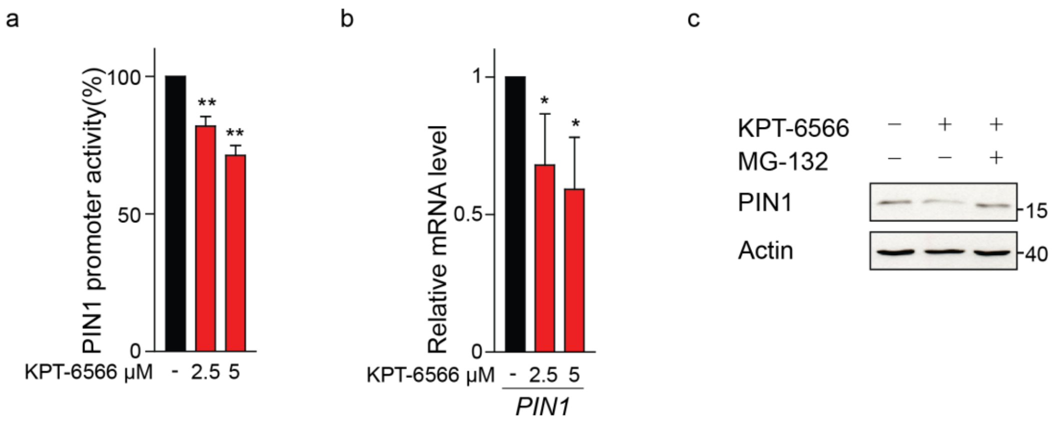

Supplementary Figure 4. (**a**) *PIN1* promoter activity in MDA-MB-231 cells treated with DMSO (-) or increasing concentrations of KPT-6566 for 48hrs. (**b**) Quantitative RT-PCR analysis in MDA-MB-231 cells treated with DMSO (-) or increasing concentrations of KPT-6566 for 48hrs. (**c**) Immunoblotting of the indicated proteins in cell lysates from MDA-MB-231 cells treated with 5 μM KPT-6566 (+), 10 μM MG132 (+) or DMSO (-) for 16hrs. Actin levels are reported as loading control, size markers are indicated. Data shown in **a**, **b** are the means ± s.d. of n=3 independent experiments, *P<0.05, **P<0.01; two-tailed Student's t-test.

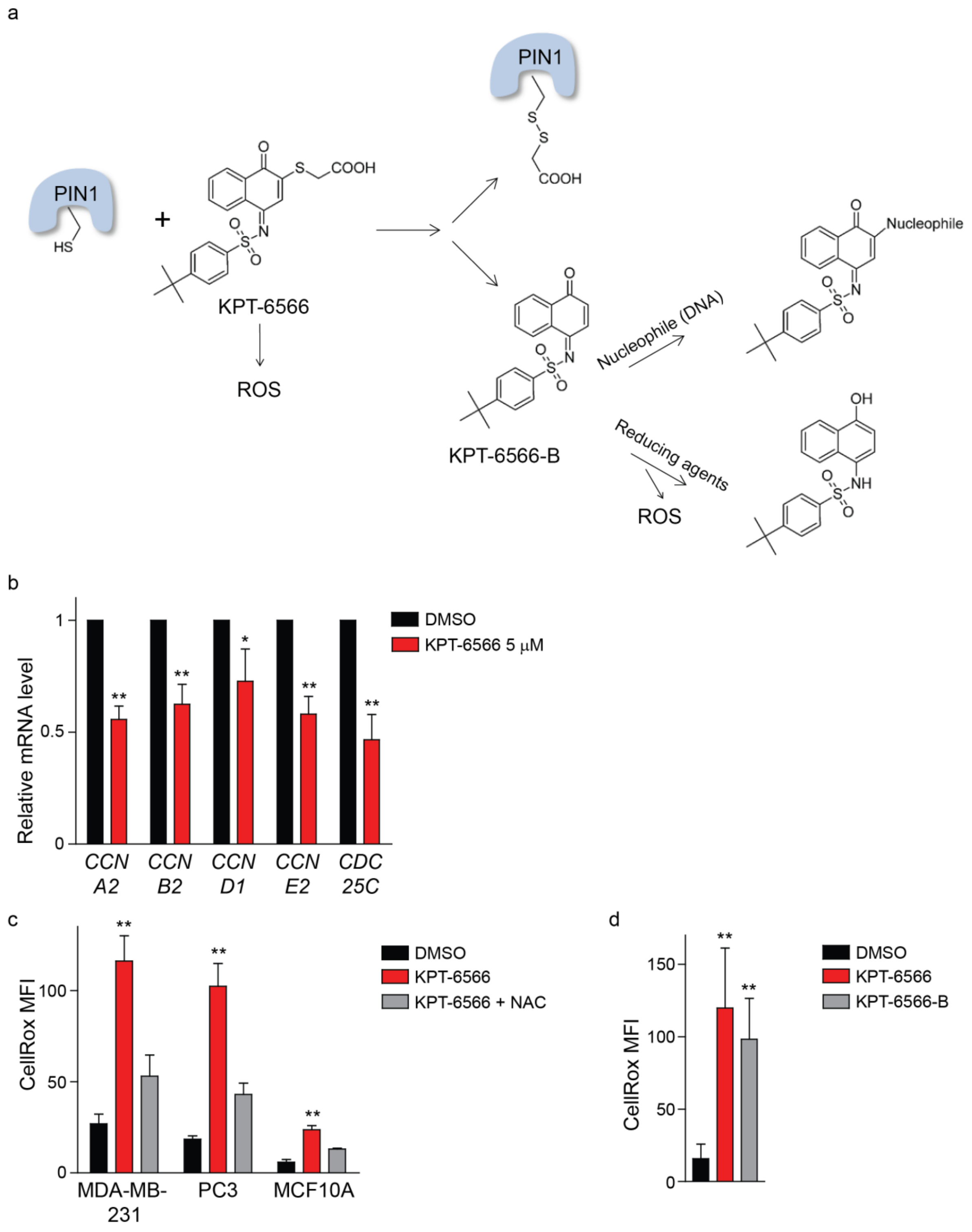

Supplementary Figure 5. (**a**) Scheme of KPT-6566 reaction with PIN1, byproduct production and possible activities of the compounds. (**b**) Quantitative RT-PCR analysis of cell cycle related genes in MDA-MB-231 cells treated with 5 μM KPT-6566 or DMSO for 48hrs. (**c**) Histogram representing CellROX mean fluorescence intensity (MFI) of MDA-MB-231, PC3 and MCF10A cells treated with 5 μM KPT-6566, 5 μM KPT-6566 plus 2.5 mM NAC or DMSO for 48hrs. (**d**) Histogram representing CellROX mean fluorescence intensity (MFI) of MDA-MB-231 cells treated

with 5 μM KPT-6566, 5 μM KPT-6566-B or DMSO for 48hrs. Data shown in **b**-**d** are the means ± s.d. of n=3 independent experiments, **P*<0.05, ***P*<0.01; two-tailed Student's *t*-test.

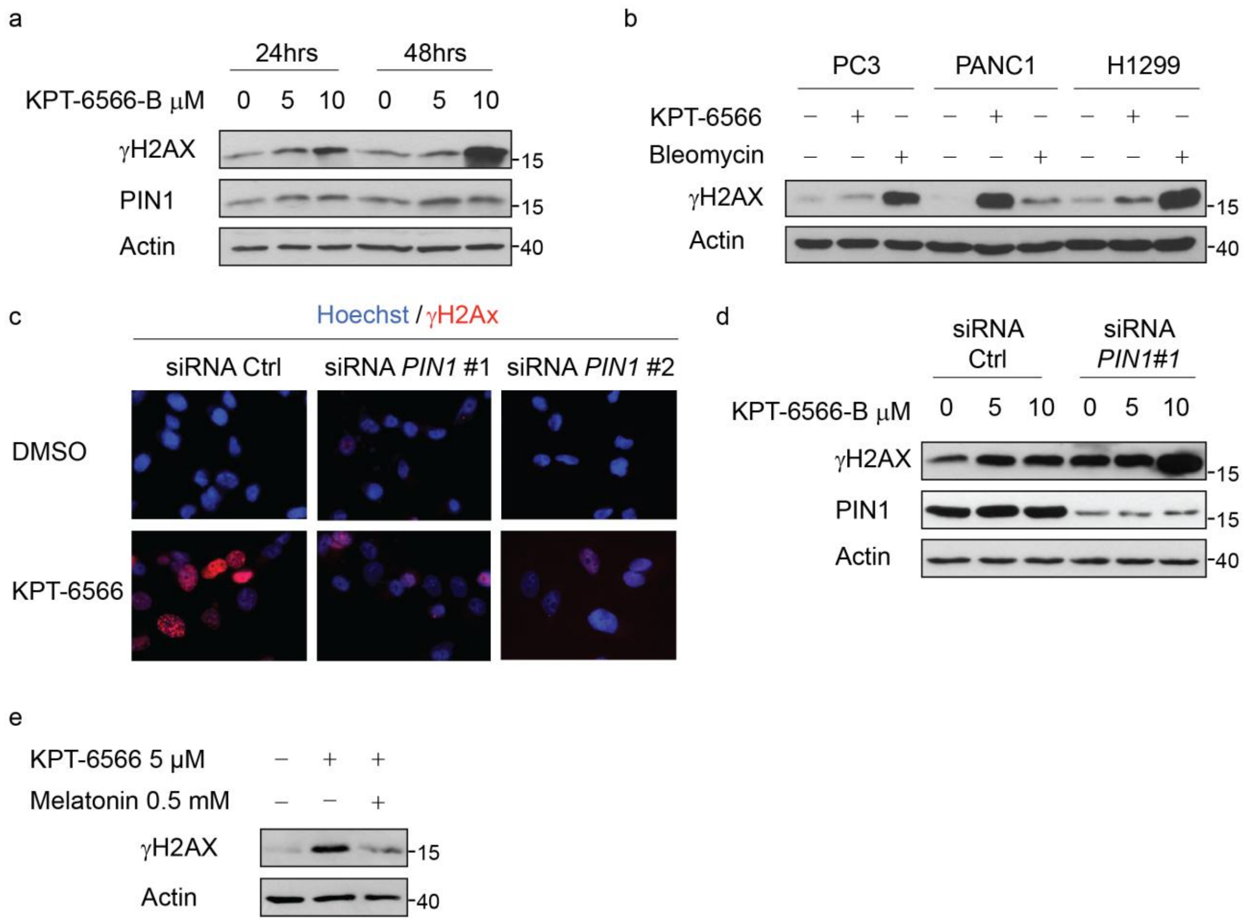

Supplementary Figure 6. (**a**) Immunoblotting of the indicated proteins from MDA-MB-231 cells treated with the indicated concentrations of KPT-6566-B or left untreated (0) and collected at the indicated time points. (**b**) Immunoblotting of the indicated proteins from PC3, PANC1 and H1299 cells respectively treated with 5 µM KPT-6566, 10 µM Bleomycin or DMSO for 48hrs. (**c**) Representative fluorescence microscope images of γH2AX (red) immunofluorescence and Hoechst (blue) staining of cells from Fig 6d. (**d**) Immunoblotting of the indicated proteins from MDA-MB-231 cells in the indicated experimental conditions. Cells were transfected with control siRNA (siRNA Ctrl) o with *PIN1* siRNA (siRNA *PIN1#1*). After 24hrs cells were trypsinized, plated and treated with the indicated concentrations of KPT-6566-B or left untreated (0) for 48hrs. (**e**) Immunoblotting of the indicated proteins from MDA-MB-231 cells treated with the indicated compounds for 48hrs. **a**,**b**,**d,e** Actin levels are reported as loading control, size markers are indicated.

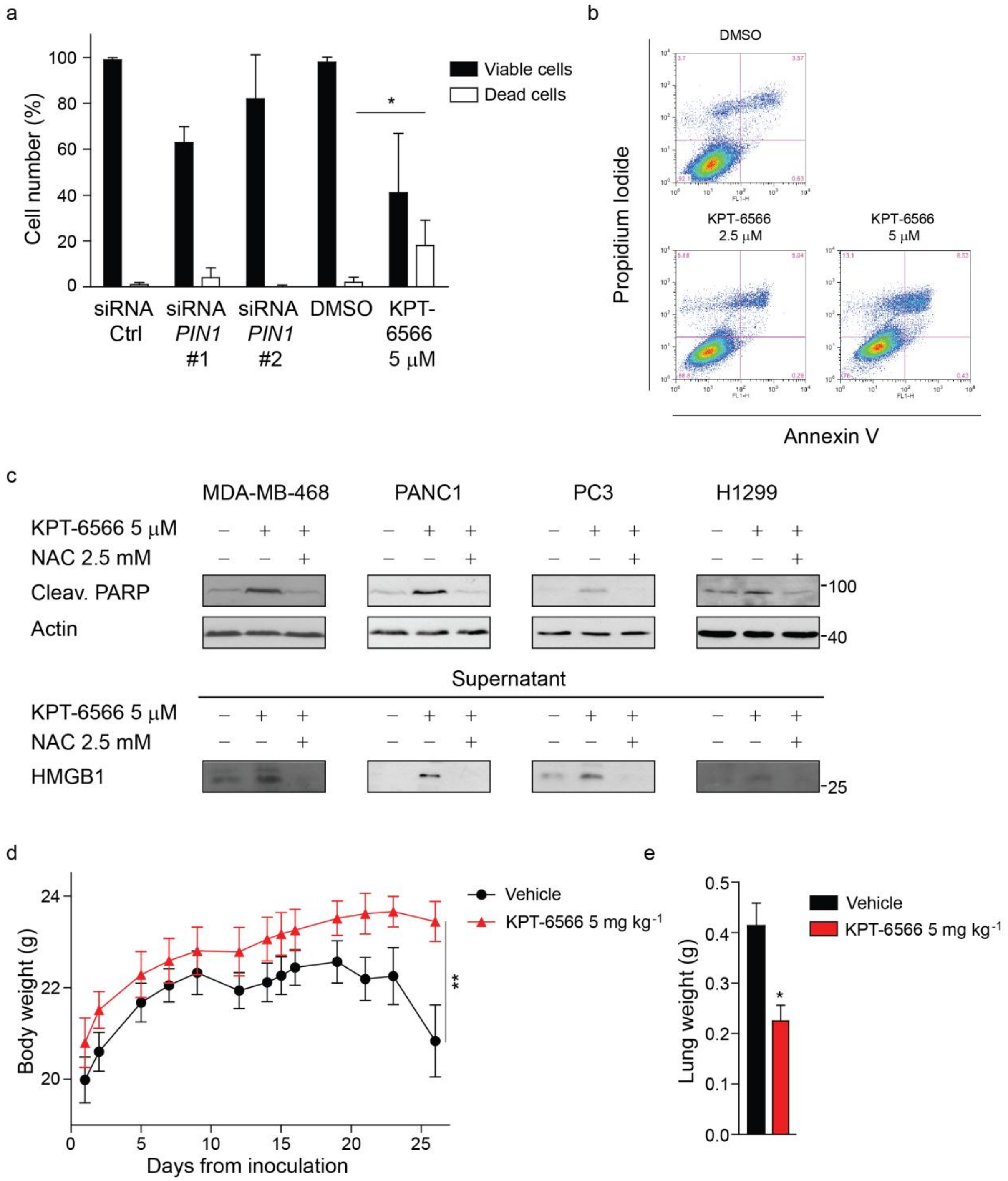

Supplementary Figure 7. (**a**) Graph showing cell number (percentage relative to siRNA Ctrl) of MDA-MB-231 cells in the indicated experimental conditions. After 48hrs of treatment, cells were trypsinized, stained with Trypan blue and counted at the optical microscope. Results are indicated as means ± s.d. of n=3 independent experiments, *P<0.05; two-tailed Student's *t*-test. (**b**) Representative FACS plot of PI/AnnexinV staining of MDA-MB-231 cells treated with DMSO or increasing amounts of KPT-6566 for 48hrs. (**c**) Top, Immunoblotting of the indicated proteins of MDA-MB-468, PANC1, PC3 and H1299 cell lysates untreated (-) or treated (+) with the indicated compounds. Bottom, Immunoblotting of HMGB1 from supernatant of the same cells as in top

panel. Actin levels are reported as loading control, size markers are indicated. (**d**) Graph showing body weight of KPT-6566 or vehicle treated mice during the period of the experiment. (**e**) Graph showing lung weight of KPT-6566 or vehicle treated mice. Data shown in **d**, **e**, are the means ± s.e.m. (n=8 for vehicle treated mice; n=7 for KPT-6566 treated mice), **P*<0.05, ***P*<0.01; two-tailed Mann-Whitney test.

Figure 2b

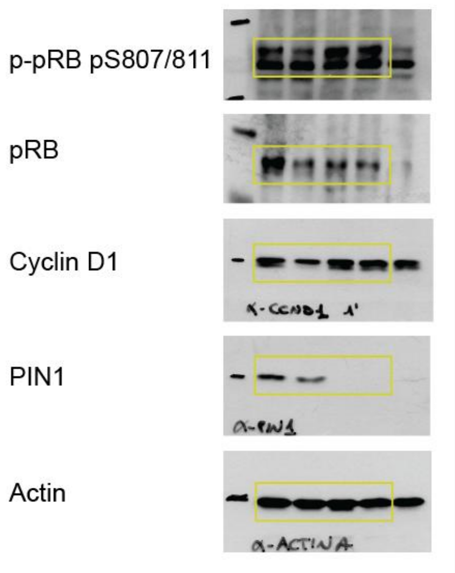

Figure 2d

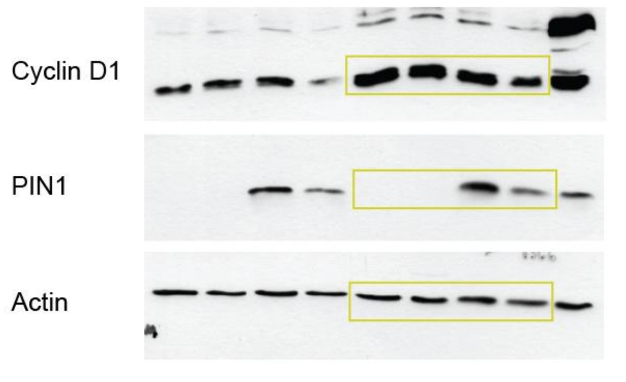

Figure 2e

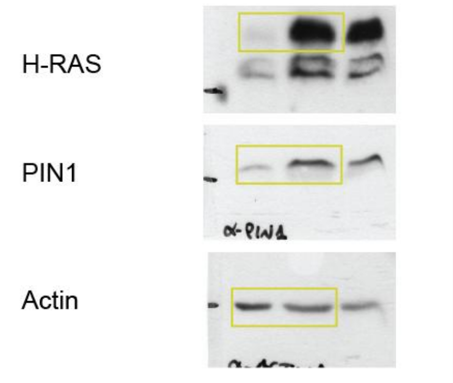

Figure 2f

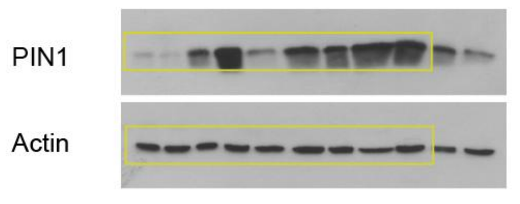

Figure 3a

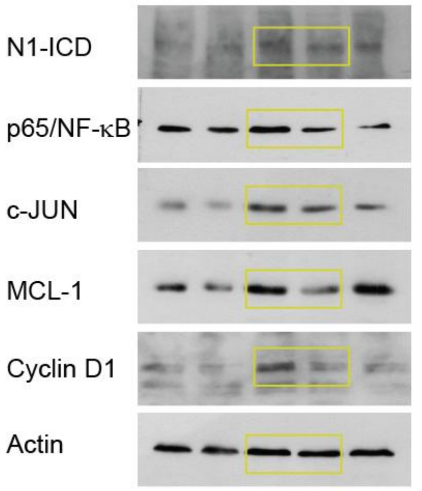

Figure 3f

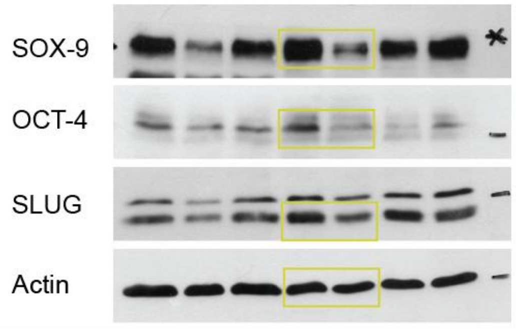

Figure 4a

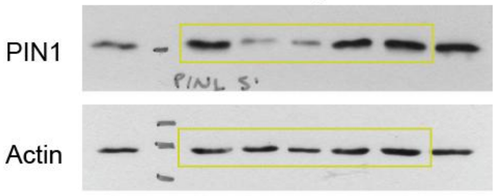

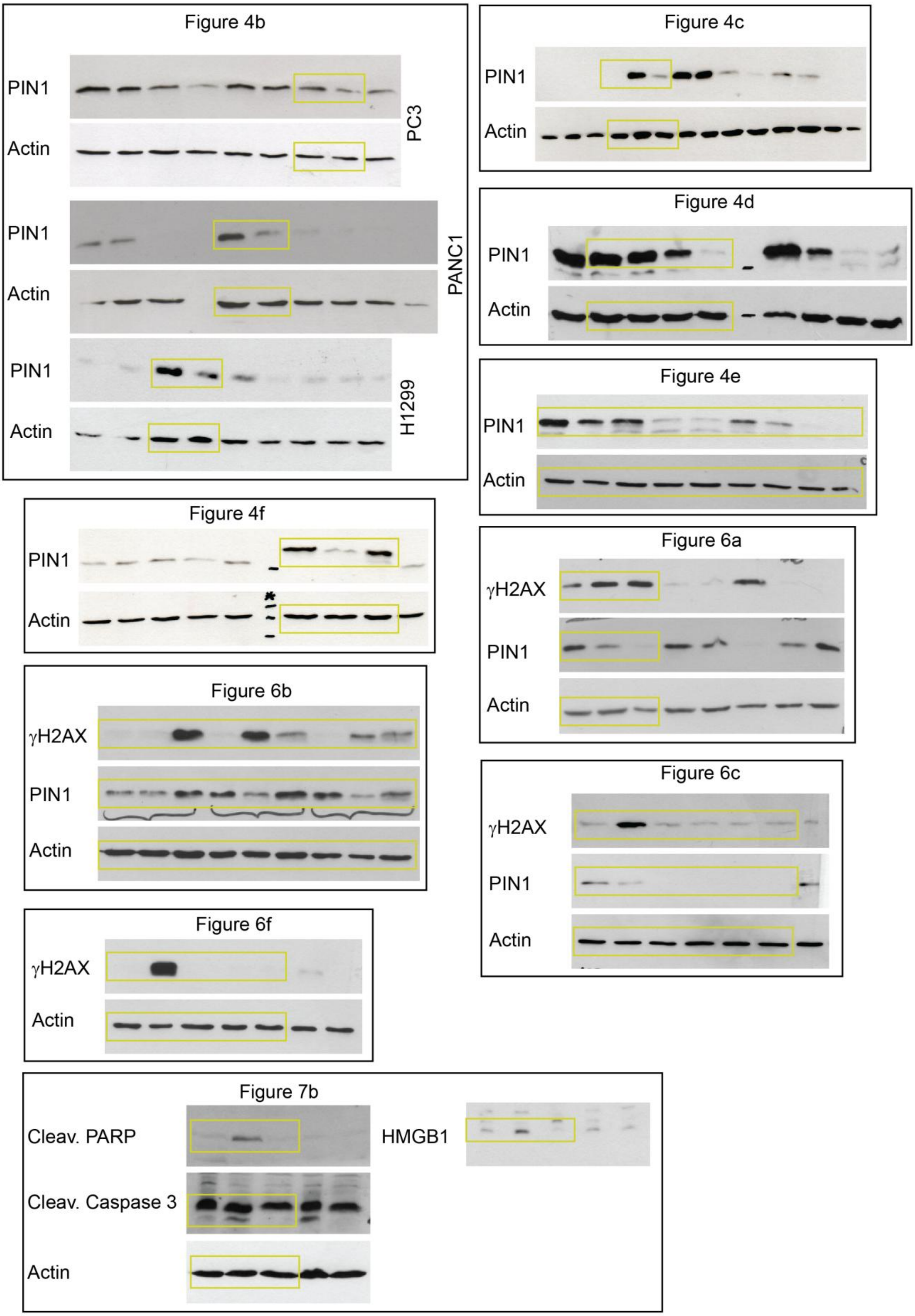

Supplementary Figure 8. Uncropped blots of figures presented in the main paper.

Supplementary Tables

Supplementary Table 1. List of IC50 values of KPT compounds used in cytotoxicity experiments performed in A375 and 3T3 cells.

	IC50 $_{A375}$ (µM)	IC50 $_{3T3}$ (µM)
KPT-6342	5.36	n.a.*
KPT-6410	8.18	20.98
KPT-6475	<0.63	6.91
KPT-6504	9.19	18.49
KPT-6517	1.75	15.67
KPT-6566	3.47	21.76
KPT-6568	n.a.	>30
KPT-6570	n.a.	>30
KPT-6605	10.28	n.a.

*n.a., not assessed

Supplementary Table 2. IC50 value of KPT-6566 for PIN1 in PPIase assay.

Inhibitor	IC50 (µM)	95% confidence intervals (µM)
KPT-6566	0.64	0.07 - 5.51

Results of n=6 independent experiments are indicated.

Supplementary Table 3. Binding poses and Glide Docking Scores of KPT-6566 docked in the PIN1 catalytic domain.

Pose	Docking Score
1	-4.280 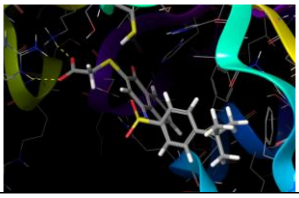
2	-4.135 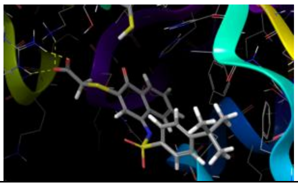
3	-4.101 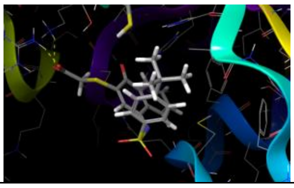
4	-3.990

	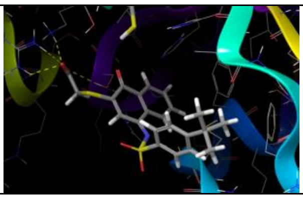
5	-3.642 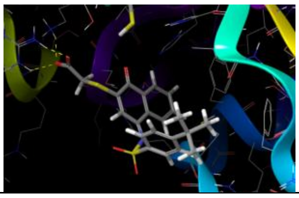
6	-3.299 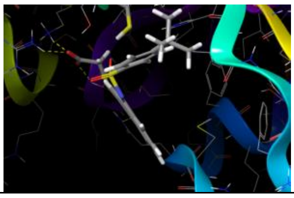
7	-3.536 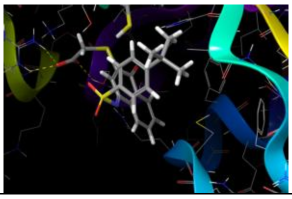

Docking scores are indicated as ligand binding free energy values. C113 and the ligands are highlighted with a licorice representation. The residues in the binding pocket are represented as lines. Dotted yellow lines indicate the presence of hydrogen bonds.

Supplementary Table 4. Estimated inactivation parameters of PIN1 by KPT-6566.

Enzyme	Inhibitor	k_{inact}	K_i	k_{inact}/ K_i
PIN1	KPT-6566	0.466 ± 0.05781 min^{-1}	625.2 ± 324.7 nM	745.4 min^{-1} nM^{-1}

Curve fitting results with std.errors of n=3 independent experiments are indicated.

Supplementary Table 5. IC50 values of KPT-6566 in viability experiments performed in MCF10A and MCF10AT1 cells.

Cell line	IC50 (µM)	95% c.i. (µM)
MCF10A	14.33	8.334 - 24.65
MCF10AT1	3.4	1.699 - 6.771

Curve fitting results with c.i. of n=3 independent experiments are indicated.

Supplementary Table 6. CD44/CD24 cell distribution analysis of MCF10A cell clones.

	vector	PIN1 + DMSO	PIN1 + KPT-6566

CD44$^+$/CD24$^-$	11.53 ± 2.54	23.9 ± 0.6	3.7 ± 0.3
CD44$^+$/CD24$^+$	85.37 ± 5.62	68.97 ± 9.26	94.77 ± 1.97
CD44$^-$/CD24$^+$	0.83 ± 0.95	2.93 ± 3.54	0.13 ± 0.06
CD44$^-$/CD24$^-$	2.3 ± 2.77	4.2 ± 5.22	1.5 ± 2.17

The percentage of cells with the indicated expression of CD44/CD24 is indicated as means ± s.d. of n=3 independent biological replicates.

Supplementary Table 7. Common downregulated genes by *PIN1* siRNA and KPT-6566.

	siRNA *PIN1*		KPT-6566	
**NAME**	**logFC**	***P* value**	**logFC**	***P* value**
LMNB1	-1.993315205	1.60E-07	-1.390713813	0.000156902
CKAP2	-1.649824429	2.73E-06	-0.753780575	0.003598795
TOP2A	-1.602626771	0.000131038	-1.078341609	0.010420359
CENPE	-1.585619226	1.76E-06	-1.039331392	0.006439969
IFRD1	-1.527953234	2.53E-05	-0.789615487	0.001657493
C4ORF46	-1.492935571	3.13E-06	-0.75996261	0.001103998
NCAPD2	-1.422371472	6.10E-06	-1.106579457	0.00019137
ASPM	-1.322426686	0.00017389	-1.079049881	0.008428549
KIF11	-1.292576165	0.000473829	-0.797084041	0.01726246
DLGAP5	-1.284419305	0.000390296	-1.252734367	0.001500357
KIF2C	-1.273094177	2.67E-05	-0.818674427	0.002214861
HNRNPM	-1.26649729	1.87E-05	-0.783694016	0.002996742
LOC727761	-1.25243774	2.89E-05	-0.798097602	0.000726711
FAM83D	-1.230971056	2.25E-05	-1.248860947	2.17E-05
AURKA	-1.215024175	3.67E-05	-1.34676716	1.98E-05
HMGB2	-1.14477821	0.000560364	-0.788573155	0.002228307
TPX2	-1.081560493	0.000129366	-1.152835417	3.52E-05
CENPA	-1.071576604	0.000106567	-0.921716931	0.000448802
C13ORF34	-1.065797926	0.00049054	-0.854864938	0.000972674
CDCA8	-1.061286474	0.00037755	-1.292188234	6.62E-06
KIF20B	-1.056059473	0.000392444	-1.070712754	0.007440821
CCNF	-1.055952082	0.000407291	-0.947015061	0.006540934
MXD3	-1.046416767	0.000302452	-1.178858625	0.001782072
HJURP	-1.036899849	0.000139923	-0.919589512	0.000422532
KIF20A	-1.011681112	0.000190637	-1.529745689	1.03E-06
NUSAP1	-0.994421483	0.001007043	-0.845747631	0.003171494
NEIL3	-0.993173905	0.000302813	-0.928656408	0.000372391
MCM3	-0.984395764	0.000297576	-0.956936429	0.000554111
CDKN2C	-0.979586993	0.001711889	-0.794034024	0.002016578
CDCA7	-0.976233578	0.000413979	-0.912224015	0.00010797
LOC643995	-0.970129977	0.000258985	-0.766798592	0.00090016
KIF4A	-0.961539576	0.000299065	-0.837655986	0.000327823
LOC100128007	-0.960067749	0.002976062	-0.805020422	0.008296733

SYNCRIP	-0.955192593	0.00220315	-0.780805854	0.012932036
SGOL2	-0.948813369	0.000514782	-0.905695274	0.006708523
HMMR	-0.948151673	0.000633793	-1.180142451	7.55E-05
GTSE1	-0.944593639	0.001028039	-0.91930718	0.000297895
MKI67	-0.943875467	0.00058885	-1.156560495	4.85E-05
KIF23	-0.930389489	0.000679496	-1.102992023	9.99E-05
C14ORF106	-0.926309045	0.000873225	-0.94092836	0.001568464
CCNA2	-0.920340126	0.000572448	-0.928737359	0.000571694
CCNB1	-0.919049598	0.000462799	-1.233733072	9.04E-05
FEN1	-0.905229686	0.000482458	-0.883205509	0.001137971
SUPT16H	-0.882711419	0.002992406	-0.765613202	0.016517827
MLF1IP	-0.858793526	0.002829338	-0.918025183	0.013001243
NASP	-0.84545566	0.000920292	-0.952505342	8.43E-05
KIFC1	-0.842754954	0.002442351	-1.222021696	0.00017394
LOC653820	-0.841801943	0.004385814	-0.958610097	0.000267962
BIRC5	-0.827335637	0.002437142	-1.19650208	5.68E-05
ESPL1	-0.803125048	0.001339697	-0.810388086	0.007067412
KIF18A	-0.795551338	0.001973556	-1.084266901	0.001282449
CDCA3	-0.767187634	0.002589411	-1.10354153	4.28E-05
CDKN3	-0.76628107	0.00453819	-1.033036632	0.000540765
CCNB2	-0.765163113	0.00189446	-0.775948086	0.000904069
LOC731314	-0.756519109	0.003534999	-0.868921232	0.00380313

Supplementary Table 8. Common upregulated genes by *PIN1* siRNA and KPT-6566.

	siRNA *PIN1*		KPT-6566	
**NAME**	**logFC**	***P* value**	**logFC**	***P* value**
AHNAK2	1.127549518	6.52E-05	0.824328418	0.000689379
ANGPTL4	0.90979909	0.00236433	1.368785676	9.10E-05
ANXA10	1.241258121	0.000924395	0.780661211	0.001895984
AVPI1	0.899366798	0.001867859	1.141838128	0.005116749
C14ORF78	1.179567691	0.000118218	1.185442274	8.25E-05
CCDC85B	0.854997268	0.004862753	1.138244481	4.24E-05
CDKN1C	0.76556803	0.00418495	1.174092299	0.000149072
CYP1B1	0.971454307	0.005609335	1.723365264	0.00010894
FBXO32	0.855254752	0.000917202	1.831448046	4.12E-07
GDF15	2.845195376	9.44E-10	1.766491436	1.52E-05
IL24	0.854560753	0.003930622	2.141642086	1.24E-06
KLF9	1.042055692	0.000808668	0.90842043	0.000415111
LOC401537	0.763206127	0.003342964	0.753039859	0.006122407
LOC653506	0.787469196	0.006057272	0.940827486	0.000647647
LY96	0.998664699	0.000218698	1.071029214	0.000394229
NRP1	0.874561843	0.000796052	1.006613622	0.000104908
PLAT	1.235653185	2.79E-05	1.155336106	0.003136798
PTPRR	0.983751456	0.000532927	1.036222539	0.000111156

RASD1	1.244988195	2.84E-05	2.037272137	0.000112903
RNF44	0.908096053	0.000906069	0.778673506	0.00202333
SERPINB2	0.890693326	0.001031445	1.750849412	6.42E-07
SPRY2	0.801291706	0.001417034	1.439677097	1.20E-05
SQSTM1	1.265910641	3.45E-05	1.126794637	2.56E-05
TFPI	0.75018459	0.013166835	0.957836867	0.000429311
TXNRD1	0.771478885	0.002945497	0.935944212	0.000800093
VASN	0.758741743	0.003248575	0.887879079	0.003360611
ZNF280D	0.994482226	0.000197028	0.907139547	0.000423506

Supplementary Table 9. Commonly enriched GO terms by *PIN1* siRNA and KPT-6566.

	siRNA *PIN1 vs.* siRNA Ctrl	KPT-6566 *vs.* DMSO
**Term**	***P*-value**	***P*-value**
GO:0000070~mitotic sister chromatid segregation	1,88E-13	2,11E-05
GO:0000087~M phase of mitotic cell cycle	1,31E-32	4,81E-17
GO:0000278~mitotic cell cycle	6,39E-37	2,55E-20
GO:0000279~M phase	2,96E-37	1,22E-16
GO:0000280~nuclear division	4,35E-33	2,77E-17
GO:0000775~chromosome, centromeric region	1,26E-15	1,40E-05
GO:0000777~condensed chromosome kinetochore	6,86E-09	0,007923592
GO:0000779~condensed chromosome, centromeric region	8,27E-12	0,002843679
GO:0005694~chromosome	1,04E-23	4,07E-09
GO:0005819~spindle	9,88E-19	6,18E-13
GO:0006259~DNA metabolic process	1,16E-11	0,001363207
GO:0006260~DNA replication	2,06E-10	0,017600514
GO:0007049~cell cycle	3,89E-40	1,87E-22
GO:0007059~chromosome segregation	3,33E-20	5,33E-07
GO:0007067~mitosis	4,35E-33	2,77E-17
GO:0007346~regulation of mitotic cell cycle	1,09E-10	2,85E-08
GO:0010564~regulation of cell cycle process	1,16E-09	2,04E-05
GO:0015630~microtubule cytoskeleton	1,32E-15	2,47E-14
GO:0022402~cell cycle process	1,05E-38	7,34E-20
GO:0022403~cell cycle phase	1,41E-42	1,12E-20
GO:0051276~chromosome organization	6,35E-10	0,000762
GO:0051301~cell division	1,25E-25	1,44E-10
GO:0051726~regulation of cell cycle	3,22E-13	3,89E-18

Supplementary Table 10. Oligonucleotides for quantitative real time PCR of human genes.

Gene symbol	Forward 5’-3’	Reverse 5’-3’
BUB1	ATTCAAGCCACAGAGTGGAGCAG	AGAACTTGTGTTGGCAACCTTAT GTG
DEPDC1	ATCAGATGACCTCCCTCACTGGGT	GGTTGCAGCAAGCCCAAAATGTT

HEY2	AGGGGGTAAAGGCTACTTTGA	TGGCGCAAGTGCTGAGATG
BIRC5	GCCCAGTGTTTCTTCTGCTT	CCGGACGAATGCTTTTTATG
PIN1	CTGGAGCTGATCAACGGCTACATCC	GCAGCGCAAACGAGGCGTCT
CCNA2	AGCAGCCTGCAAACTGCAAAGTTG	TGGTGGGTTGAGGAGAGAAACAC
CCNB2	GGCTGGTACAAGTCCACTCC	GAAGCCAAGAGCAGAGCAGT
CCND1	ACGGCCGAGAAGCTGTGCATC	CCTCCGCCTCTGGCATTTTGGAG
CCNE2	TGAGCCGAGCGGTAGCTGGT	GGGCTGGGGCTGCTGCTTAG
CDC25C	GTATCTGGGAGGACACATCCAGGG	CAAGTTGGTAGCCTGTTGGTTTG
P21	TACCCTTGTGCCTCGCTCAG	GAGAAGATCAGCCGGCGTTT
BCL2	GCGACTCCTGATTCATTGG	GTCTACTTCCTCTGTGATGTTG
GADD45	GTGGTGTTGTGCCTGCTG	AGGATGTTGATGTCGTTCTCG
cFOS	GGGGCAAGGTGGAACAGTTA	AGTTGGTCTGTCTCCGCTTG
HO1	AAGACTGCGTTCCTGCTCAA	GGGGGCAGAATCTTGCACTT
NQO1	TGAAAGGCTGGTTTGAGCGA	CTGCCTTCTTACTCCGGAAGG
TXNRD1	CTTTTTCATTCCTGCTACTCTACC	CTCTCTCCTTTTCCCTTTTCC
DNAJB9	TAGTCGGAGGGTGCAGGATA	CGCTCTGATGCCGATTTTGG
H3	GTGAAGAAACCTCATCGTTACAGGCCTGGT	CTGCAAAGCACCAATAGCTGCACTCTGGAA

Supplementary Table 11. Oligonucleotides for cloning.

Original vector	Forward 5'-3'	Reverse 5'-3'
pcDNA3HA PIN1; pcDNA3HA PIN1 S67E; pcDNA3HA PIN1 C113A	TACTTCCAATCCATGGCGGACGAGGAGAAGCTGCC	TATCCACCTTTACTGTCATCACTCAGTGCGGAGGATGATGTG
pANT7_cGST FKBP4	CATCATAGATCTATGACAGCCGAGGAGATGAAG	CATCATCTCGAGCTATGCTTCTGTCTCCACCTG
pANT7_cGST PPIA	CATCATGGATCCATGGTCAACCCCACCGTGTTC	CATCATCTCGAGTTATTCGAGTTGTCCACAGTC

Supplementary Table 12. siRNA sequences.

siRNA	Sequence 5'-3'	Reference
siRNA *PIN1*#1	CGGGAGAGGAGGACUUUGA	[7]
siRNA *PIN1*#2	GCCAUUUGAAGACGCCUCG	[7]
siRNA Ctrl	Allstar NEGATIVE control, Qiagen	

Supplementary Methods

Recombinant protein production and purification

Recombinant human PIN1 (Uniprot ID: Q13526), PIN1 S67E, and PIN1 C113A were cloned in frame in pNIC28-Bsa4 vector (generous gift from Opher Gileadi, SGC Oxford) by Ligase Independent Cloning (LIC) as fusion proteins with an N-terminal 6xHis tag followed by TEV cleavage site starting from pcDNA3 expressing vectors[1–3]. Protein expression was done in *Escherichia coli* BL21(DE3) cells using the autoinduction protocol[4] incubating cells at 37°C for 4 hours followed by 18 hours incubation at 25°C. Soluble proteins were purified from the total cellular lysate by binding to Ni-NTA Agarose (Qiagen) and eluting with 50 mM Tris pH 8.0, 500 mM NaCl, 500 mM Imidazole. Proteins were buffer exchanged in 50 mM Tris pH 8.0, 150 mM NaCl, 0.5 mM EDTA, 1 mM DTT and the 6xHis tag removed by 6xHisTEV protease (produced in house) performed during o/n dialysis at 4°C. The uncleaved proteins and the 6xHis-TEV were removed by affinity chromatography on Ni-NTA Agarose, and untagged PIN1 (WT and mutants) was collected in the flowthrough fraction and further purified on Superdex 75 10/300 size exclusion column (GE Healthcare) using 30 mM HEPES pH 7.8, 100 mM NaCl as mobile phase. Finally the proteins were dialyzed at 4°C for 18 hours in 30 mM HEPES pH 7.8 to remove trace of salt.

Human FKBP4 (Uniprot ID: Q02790) and PPIA (Uniprot ID: P62937) were cloned in frame with the pGTVL1 vector (generous gift from Opher Gileadi, SGC Oxford) by Ligase Independent Cloning (LIC) as fusion proteins with an N-terminal GST starting from pANT7_cGST FKBP4 and PPIA expressing vectors. The proteins were expressed in E. coli BL21(DE3) cells grown at 37°C in Terrific Broth medium (Fluka) supplemented with 1% (w/v) Glycerol (Sigma) and induced with 1 mM isopropyl thio-β-galactoside (Sigma) at midlog growth phase. The cell cultures were collected by centrifugation after 3 h growth at 37°C. Soluble GST-FKBP4 and GST-PPIA were affinity purified from the total cellular lysate on Protino® Glutathione Agarose 4B (Macherey-Nagel) and eluted with 20 mM reduced glutathione (Sigma) in 20 mM Tris pH 8.0, 10 mM DTT. GST fusion proteins were further purified by size exclusion chromatography on HiLoad 16/60 Superdex 200 column (GE Healthcare) in 30 mM HEPES pH 7.8, 100 mM NaCl, 2 mM DTT. Finally the recombinant proteins were buffer exchanged (in 30 mM HEPES pH 7.8, 10 % Glycerol) and concentrated on AMICON15 devices (Millipore), using 50 kDa MWCO for GST-FKBP4 and 10 kDa MWCO for GST-PPIA.

Synthesis of compounds 1 – 3.

Compound **1** was obtained through a modified procedure previously described for similar structures[5]. Briefly, intermediate **3** was synthetized in 62 % yield via regioselective[5] coupling

reaction between 2-chloro-1,4-naphthoquinone and 4-*tert*-butylbenzene-1-sulfonamide using titanium (IV) chloride tetrahydrofuran complex and trimethylamine (TEA). The same conditions were applied also for the preparation of the compound **2**. Starting from naphthalene-1,4-dione, the procedure afforded, besides the title compound **2** (yield 23 %), also the chloro derivative **3** as sideproduct. This fact underlines the electrophile nature of compound **2**, being reasonably able to react with chlorine ions released from $TiCl_4$. In the following step, the intermediate **3** was then subjected to nucleophilic substitution by thioglycolic acid in the presence of dry pyridine to afford the desired acid **1** in 64 % yield.

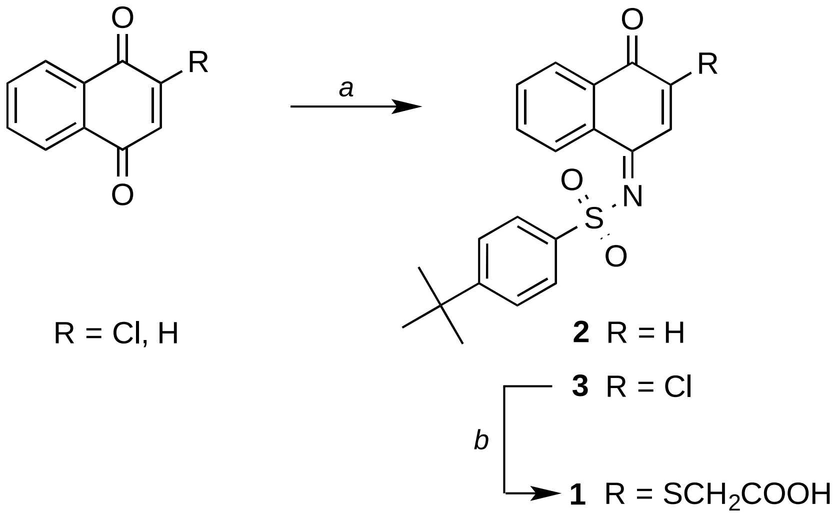

Scheme 1. (**a**) 4-*tert*-butylbenzene-1-sulfonamide, $TiCl_4$·2THF, TEA, dry THF, reflux; (**b**) $HSCH_2COOH$, dry pyridine, dry THF, rt.

General methods: All chemical reagents were obtained from commercial sources (Sigma Aldrich, Alfa Aesar) and used without further purification. Thin-layer chromatography (TLC) was carried out to monitor the process of reactions. Analytical grade solvents (diisopropyl ether, dichloromethane [DCM], ethyl acetate, methanol [MeOH], petroleum ether b.p. 40 - 60°C [petroleum ether], hexane) were used without further purification. Tetrahydrofuran (THF) was distilled immediately prior to use from Na and benzophenone under N_2. Purification of compounds was achieved with flash column chromatography on silica gel (Merck Kieselgel 60, 230-400 mesh ASTM) using the eluents indicated. The biological experiments were conducted on compounds with a purity of at least 95%. Purity was determined by HPLC analyses, performed on a Waters HPLC system composed by: Waters 1525EF binary pump, Waters 717 plus autosampler and Waters 2996 PDA detector. The analytical column was Waters XTerra Phenyl (4.6x150, 5um) column, flow 1 ml/min; compounds were dissolved in CH_3CN or MeOH. The mobile phase consisted of MeOH (or

CH_3CN)/water with 0.1% trifluoroacetic acid; two gradient profiles of mobile phase were used to assay the purity of each compound.

Melting points (m.p.) were measured on a capillary apparatus (Büchi 540). The final m.p. determination was achieved by placing the sample at a temperature 10° C below the m.p. and applying a heating rate of 2° C min^{-1}. MS spectra of compound **3** was performed on Waters Micromass ZQ equipped with ESCi source for electrospray ionization. ^{1}H- and ^{13}C-NMR spectra for all described compounds were performed on a Bruker Avance 300 instrument. For coupling patterns, the following abbreviations are used: br = broad, s = singlet, d = doublet, dd = doublet of doublets, t = triplet, q = quartet, m = multiplet. Chemical shifts (δ) are given in parts per million (ppm). For compounds **1** and **2**, HRMS spectra were recorded on an LTQ Orbitrap mass spectrometer (Thermo Scientific, Bremen, Germany) equipped with an atmospheric pressure interface and an ESI ion source instrument.

4-*tert*-Butyl-*N*-(3-chloro-4-oxonaphthalen-1(4*H*)-ylidene)benzene-1-sulfonamide (3). $TiCl_4$ ·2THF (520 mg, 1.56 mmol) and TEA (0.476 mL, 3.43 mmol) were added to a solution of 2-chloro-1,4-naphthoquinone (300 mg, 1.56 mmol) and 4-*tert*-butylbenzene-1-sulfonamide (333 mg, 1.56 mmol) in dry THF (15 mL) at 0 °C. The mixture was retrieved to room temperature, then heated at reflux for 3h. The cooled black mixture was poured into ethyl acetate (100 mL) and the resulting suspension was filtered through a pad of Celite. The filtrate was concentrated, the residue obtained was suspended in DCM (100 mL). The brown insoluble particles were removed by filtration, and the filtrate was again concentrated to dryness. The residue was purified by flash chromatography (eluent: petroleum ether/ethyl acetate 95/5 v/v) to obtain a yellow solid (mp: 194.9 - 196.0°C from crystallization by DCM/hexane). Yield: 62 %; TLC (petroleum ether/ethyl acetate, 90:10 v/v): R_f = 0.43; ^{1}H NMR (300 MHz, $CDCl_3$): δ 8.69 (s, 1H), 8.20 – 8.16 (m, 2H), 8.00 (d, *J* = 8.5 Hz, 2H), 7.77 – 7.57 (m, 4H), 1.38 (s, 9H). ^{13}C NMR (75 MHz, $CDCl_3$) δ 177.14, 160.80, 157.71, 145.05, 137.29, 134.22, 133.94, 133.08, 131.46, 129.77, 127.94, 127.49, 127.02, 126.34, 35.45, 31.20. MS (*m*/*z*): $[M]^+$ 388.

2-{[4-(4-*tert*-Butylbenzenesulfonamido)-1-oxo1,4-dihydronaphthalen-2-yl]sulfanyl}acetic acid (1). Compound **3** (150 mg, 0.39 mmol) was dissolved in THF (10 mL) and mercaptoglycolic acid (0.27 mL, 0.39 mmol) and dry pyridine (0.31 mL, 0.39 mmol) were added to the resulting solution. The mixture was stirred at room temperature for 30 min, then concentrated under reduced pressure. The orange residue was dissolved in ethyl acetate, the resulting solution was washed with 0.5 N HCl, water and brine, dried over Na_2SO_4 and concentrated under reduced pressure. The crude material was purified by flash chromatography (eluent: DCM/ethyl acetate/HCOOH 80:20:0.1 v/v/v) to give a yellow solid. Yellow solid (mp: 184.3-187.4 °C from trituration with diisopropyl

ether). Yield 64 %. TLC (DCM/MeOH/HCOOH, 95:5:0.1 v/v/v): R_f = 0.22; ^{1}H NMR (300 MHz, DMSO-d_6): δ 13.38 (br s, 1H), 8.13 - 8.02 (m, 2H), 7.98 (d, J = 8.4 Hz, 2H), 7.88 – 7.77 (m, 3H), 7.71 (d, J = 8.4 Hz, 2H), 3.97 (s, 2H), 1.33 (s, 9H). ^{13}C NMR (75 MHz, $CDCl_3$) δ 180.42, 175.36, 168.48, 159.80, 156.75, 153.47, 137.71, 134.72, 133.78, 132.89, 131.25, 126.90, 126.82, 126.45, 119.75, 35.03, 33.32, 30.75. HRMS (m/z): $[M]^+$ calcd. for $C_{22}H_{21}NO_5S_2$, 444.0939; found, 444.0934.

4-*tert*-Butyl-*N*-(4-oxonaphthalen-1(4*H*)-ylidene)benzene-1-sulfonamide (2). This compound was prepared using the procedure described for compound **1** except for using naphthalene-1,4-dione (247 mg, 1.56 mmol) as starting material. After filtration, the crude material was purified by two subsequent flash chromatography steps (eluents: petroleum ether/ethyl acetate 95/5 v/v and petroleum ether/DCM 50:50 v/v) to obtain a yellow solid (mp: 183.9-185.0°C; from trituration with diisopropyl ether). Yield: 23 %. TLC (petroleum ether/DCM 50:50 v/v): R_f = 0.25; ^{1}H NMR (300 MHz, $CDCl_3$): δ 8.44 (d, J = 10.5 Hz, 1H), 8.18 (d, J = 7.5 Hz, 1H), 8.12 (d, J = 7.7 Hz, 1H), 8.00 (d, J = 8.5 Hz, 2H), 7.79 - 7.64 (m, 2H), 7.61 (d, J = 8.5 Hz, 2H), 6.89 (d, J = 10.5 Hz, 1H), 1.37 (s, 9H). ^{13}C NMR (75 MHz, $CDCl_3$) δ 184.00, 163.08, 157.50, 137.61, 136.96, 133.81, 133.66, 133.26, 132.44, 131.91, 127.42, 126.85, 126.76, 126.27, 35.43, 31.22. HRMS (m/z): $[M]^+$ calcd. for $C_{20}H_{19}NO_3S$, 354.1158; found, 354.1162.

Generation of PIN1 knockout MDA-MB-231 cells

PIN1 knockout MDA-MB-231 cells were generated by CRISPR/Cas9 genome engineering technology. Briefly, to minimize off-target effects and increase indel efficiency the all-in-one Nickase-Ninja® construct pD1401-AD: CMV-Cas9N-2A-GFP, Cas9-ElecD was employed expressing a Cas9 nickase mutant and Green-fluorescent protein (GFP) and harboring two tandem gRNAs for human *PIN1* (gRNA for Hs:PIN1:9,949,169 - 9,949,210) on a single vector (DNA2.0)[6]. MDA-MB-231 cells were transfected and cells expressing high levels of GFP were recovered by sorting performed on ARIA II cell sorter (Becton Dickinson) and plated at clonogenic density. Single clones were grown and derived protein lysates analyzed by western blot for PIN1 protein expression. Genomic DNA of one clone lacking PIN1 expression was extracted, and the *PIN1* locus containing the region of Exon2 (targeted by the gRNAs) was PCR amplified. Amplicons representing the two modified alleles that migrated at different molecular weights in agarose gel electrophoresis were purified and checked by sequencing (Eurofins Genomics) to detect indels justifying absence of PIN1 protein expression. Sequencing unveiled one deletion of two nucleotides and one insertion of 17 nucleotides, respectively, that led in both cases to a frameshift (FS) resulting

in the genotype *PIN1*FS_asn40/FS_gln49. *PIN1* RNA levels were very low compared to control clones, indicating degradation through the non-sense RNA decay pathway.

Plasmids, Retroviral Vectors

Retroviral pLPC-HA-PIN1, pMSCV-HA-PIN1 and pSR shRNA *PIN1* (corresponding to the siRNA sequence #1) were already described[1,7].

Oligonucleotides

All oligonucleotides were supplied by MWG and are listed in Supplementary Tables 10-12.

Antibodies for western blot, immunofluorescence, and flow cytometric analysis

The following antibodies were used: rabbit polyclonal anti-PIN1 (home-made, 1:1000)[2], mouse anti-pRB (554164, BD Pharmigen, 1:1000), rabbit polyclonal anti-p-pRB pS807/811 (9308, Cell Signaling, 1:1000), rabbit polyclonal anti-Cyclin D1 (2922, Cell Signaling, 1:1000), mouse monoclonal anti-Cyclin D1 (2926, Cell Signaling, 1:1000), rabbit polyclonal anti-Actin (A2066, Sigma, 1:2000), rabbit polyclonal anti-H-RAS C-20 (sc-520, Santa Cruz, 1:500), rabbit polyclonal anti-Cleaved Notch1 Val1744 (4147, Cell Signaling, 1:500), rabbit polyclonal anti-NF-κB p65 C-20 (sc-372, Santa Cruz, 1:10000), rabbit polyclonal anti-c-JUN 60A8 (9165, Cell Signaling, 1:1000), rabbit polyclonal MCL-1 S-19 (sc-819, Santa Cruz, 1:500), rabbit polyclonal anti-Oct4 (2750, Cell Signaling, 1:1000), rat anti-HA (11867423001, Roche, 1:1000), rabbit monoclonal anti-Slug C19G7 (9585, Cell Signaling, 1:1000), rabbit polyclonal anti-Sox-9 H-90 (20095, Santa Cruz, 1:1000), mouse monoclonal anti-γH2AX Ser139 clone JBW301 (05-636, Millipore, 1:1000 for western blot analysis, 1:240 for immunofluorescence analysis), rabbit polyclonal anti-HMGB1 (ab18256, Abcam, 1:1000), rabbit monoclonal anti-Cleaved Caspase-3 Asp175 5A1E (9664, Cell Signaling, 1:500), rabbit polyclonal anti-Cleaved PARP p85 Fragment (G7341, Promega, 1:1000). The following secondary antibodies were used: anti-mouse IgG-Peroxidase antibody produced in goat (A4416, Sigma, 1:2000), anti-rabbit IgG-Peroxidase antibody produced in goat (A6154, Sigma, 1:2000), anti-rat IgG-Peroxidase antibody produced in goat (A9037, Sigma, 1:2000), goat anti-mouse IgG Alexa Fluor 568 (A-11004, Life Technologies, 1:500).

Flow cytometric analyses were performed with mouse anti-human PE conjugated anti-CD44 and FITC conjugated anti-CD24 antibodies (550989 & 555427 BD Biosciences, 1:200) and APC coniugated anti-CD326 (EpCAM) clone HEA-125 (130-098-118, Miltenyi Biotech, 1:50).

Supplementary References

1 Rustighi A, Zannini A, Tiberi L, Sommaggio R, Piazza S, Sorrentino G *et al.* Prolyl-isomerase Pin1 controls normal and cancer stem cells of the breast. *EMBO Mol Med* 2014; **6**: 99–119.

2 Rustighi A, Tiberi L, Soldano A, Napoli M, Nuciforo P, Rosato A *et al.* The prolyl-isomerase Pin1 is a Notch1 target that enhances Notch1 activation in cancer. *Nat Cell Biol* 2009; **11**: 133–142.

3 Zacchi P, Gostissa M, Uchida T, Salvagno C, Avolio F, Volinia S *et al.* The prolyl isomerase Pin1 reveals a mechanism to control p53 functions after genotoxic insults. *Nature* 2002; **419**: 853–857.

4 Studier FW. Protein production by auto-induction in high-density shaking cultures. *Protein Expr Purif* 2005; **41**: 207–234.

5 Ge Y, Kazi A, Marsilio F, Luo Y, Jain S, Brooks W *et al.* Discovery and synthesis of hydronaphthoquinones as novel proteasome inhibitors. *J Med Chem* 2012; **55**: 1978–1998.

6 Ran FA, Hsu PD, Lin CY, Gootenberg JS, Konermann S, Trevino AE *et al.* Double nicking by RNA-guided CRISPR cas9 for enhanced genome editing specificity. *Cell* 2013; **154**: 1380–1389.

7 Girardini JE, Napoli M, Piazza S, Rustighi A, Marotta C, Radaelli E *et al.* A Pin1/mutant p53 axis promotes aggressiveness in breast cancer. *Cancer Cell* 2011; **20**: 79–91.
